# Teaching Nature and Architecture: Student-Led Account of Biomimicry Innovations in the Tropics

**DOI:** 10.3390/biomimetics8010013

**Published:** 2023-01-01

**Authors:** Girirajan Arumugam, Siti Norzaini Zainal Abidin, Camelia May Li Kusumo, Anuj Jain

**Affiliations:** 1 School of Architecture, Building and Design, Taylor’s University, Subang Jaya 47100, Malaysia; 2 Biomimicry Singapore Network and bioSEA Pte. Ltd., 68 Chestnut Avenue, Singapore 679521, Singapore

**Keywords:** biomimicry design, built environment, day lighting, structure making, thermoregulation, water management, ventilation

## Abstract

The built environment has a huge carbon footprint, and decarbonizing it is essential in driving our sustainability efforts. We take the approach of biomimicry by working with Master of Architecture students from Taylor’s University in Malaysia. The students partake in a 14-week Nature and Architecture design module at the university where they develop biomimicry solutions for the built environment with a focus on sustainability. The students undergo a three-step process of scoping the design problem in the tropical climate and urban context, researching the biological literature, abstracting design ideas, and finally, developing prototypes. The module presents an opportunity for students to study nature and immerse in experiential learning in the megadiverse geographies of Malaysia and wider tropical southeast Asia. This paper describes the student works developed in various module runs from 2017 to 2022 under the supervision of the authors. Selected student projects were analyzed thematically, curated, and classified by frequently occurring themes. Finally, their design implications and challenges faced are presented. We found the following five themes to be most commonly chosen by the students—thermoregulation, structure making, water management, daylighting and ventilation, and transport and mobility. Lastly, we also conducted postgraduation student surveys on their learnings from the module. Through our synthesis, we discuss how student works can bridge the gap of applying biomimicry into practice and the limitations thereof in mainstreaming the practice in the built environment of tropical southeast Asia.

## 1. Introduction

The built environment consumes over a third of global energy demand and is responsible for at least a third of global greenhouse gas emissions [1,2]. Urgent measures should be implemented to mitigate its greenhouse gas emissions. Natural organisms are adapted to context and have highly developed place-based and efficient strategies. These strategies when emulated, i.e., through biomimicry (bios: life, mimesis: imitation), can lead to energy efficient solutions and, thereby, reduce the built environment’s greenhouse gas emissions. Biomimicry uses the 3-M approach of nature: model, measure, and mentor [3]. Freely available resources such as https://asknature.org (accessed on 29 November 2022) provide a rich repository of ideas from nature to mimic. The limitation is that such resources are generic and not tailored for built-environment applications.

Architecture practitioners and students have been inspired by nature and found new ways to tackle design challenges that relate to structures, natural ventilation and lighting, thermal regulation, and environmental issues such as air pollution, noise pollution, water management, and soil erosion [4]. They look to organisms and their strategies that help them thrive in similar environmental contexts and face the same abiotic conditions [5]. Following the principle of “form follows function”, living organisms have unique forms and geometries that enable them to function, adapt to, and survive stressors posed by the outdoor environment. These geometries and forms can be mimicked to create building forms and structures that are adapted to place [3]. As an example, the Gherkin tower in London designed by Norman Foster took inspiration from the lattice-like exoskeleton structure of the Venus’ flower basket sponge (*Euplectella aspergillum*) and applied it to the building structure that serves to reduce wind deflections [6]. Efficient thermoregulation of buildings can be achieved by improving ventilation, managing heat gain, and humidity control through building envelopes. The Eastgate Centre building in Harare, Zimbabwe, took inspiration from termite mound ventilation and created a highly porous building skin that moderates the temperature difference between the indoor and outdoor environment [7]. The National Aquatics Center in Beijing, designed by PTW Architects and Arup, took inspiration from soap bubbles to subdivide the space in the building with optimized lighting via building facades. This reduced energy consumption by 30% and reduced the artificial lighting by 55% [8].

The main aim of this paper is to showcase the diversity of ideas developed by architectural students through a Nature and Architecture (N&A) module, their learning outcomes, and how their knowledge was applied to the industry after the students’ graduation. This is achieved through a showcase of student projects where biomimicry concepts are presented classified by architectural themes. Student responses from a questionnaire on learning outcomes and biomimicry applications are presented.

Biomimicry design studios and modules for architecture students are now run worldwide such as in Egypt [9], New Zealand [10], Turkey [11], and USA [12,13]. Studies have highlighted the importance of recommending biomimicry in architecture as a core subject to raise awareness among architectural students and guide them to pursue biomimicry in their professional practice [14]. It has been shown that student exercises in biomimicry can foster their creativity and help in understanding the process of learning from nature and applying it to the architectural design process [15].

However, such initiatives are far fewer in southeast Asia (except see digital biomimetics studio in Singapore [16], and the biomimicry design program in Taiwan [17]. Malaysia, and the wider southeast Asian region, is well known for its megadiverse biodiversity where such modules provide an opportunity for students to immerse in nature study. They can become avenues for students to discover champion species that may provide innovative solutions to place-based architectural problems for the tropical climate.

Mimicking ecosystem processes may be complex and difficult for designers, but mimicking ecosystem functions may be easier as many aspects are measurable [18]. Architectural modules have also shown that mimicking nature’s forms especially when it involves non-Euclidean geometry is complex and that students may sometimes struggle [19]. This challenge can be overcome if students use simulation, material-based thinking, and computer-aided design tools [19], which was encouraged but not compulsory in the N&A module.

The Nature and Architecture N&A ARC70803 Module at Taylor’s University in Malaysia has been offered as an elective for postgraduate architectural students since March 2018 by the School of Architecture, Building, and Design. Inspired by the Malaysian eco-architect Dr. Ken Yeang’s work on bio-climatic design and ecomimicry, the module aimed at exposing students to sustainability in architecture through the lens of biomimicry. We hypothesized that this approach would improve student creativity and widen their horizons by applying biomimicry theory to built-environment issues through an engaging teaching and learning experience.

## 2. Materials and Methods

### 2.1. Thematic Analysis and Survey Questionnaires

The research methodology employed was to review the result of the student’s works and the impact of the biomimicry learning on their own architecture practice. Therefore, two different qualitative research methodologies were employed:

1. Thematic analysis of 69 student works between 2017 and 2022 was done. Of these, selected student projects were curated, coded systematically, and classified by frequently occurring themes in building design such as thermoregulation and ventilation. Themes were identified based on the frequency of choice by the students in their application of biomimicry concepts. Projects were also classified based on the type of biomimicry—form, process, or system [3].
2. A survey questionnaire was floated to students of year 2017 to 2022, in which we collected feedback from 28 students on their learning experience and the possible application of biomimicry at their own architecture practice. The contents of the questionnaire included an introduction and the purpose of the questionnaire followed by targeted questions. The survey was conducted after the students had graduated from their study and were typically working at architecture firms. An online questionnaire was used as the students were working at physically different locations. The survey consisted of four sections: reason to choose the module, learning experience, feedback on the module, and industry application of biomimicry learning.

Ethical procedures were followed in this research. Informed consent to participate was taken from the students. The anonymity of survey questionnaire participants was maintained.

### 2.2. Biomimicry Teaching and Learning Philosophy

Using the philosophy of nature as an idea generator, the N&A module accentuates the process of studying and understanding nature along with its context-based adaptations. A *Design to Biology* approach was used in which students looked to nature for solutions to built-environmental problems of their interest. Students were free to design biomimetic products or services as part of their 14-week individual project that met the needs of the built-environment industry by mimicking form, process, or systems. 

The teaching and learning approach for the module was lecture and tutorial based, with students engaging in hands-on and hybrid (online and in-person) learning experiences. The module integrated the use of in-person and online materials, tools, and activities to amplify the interactions between teachers and learners. Online materials included YouTube videos, TED talks, workshops, subjective questions, discussions, and journals. During the COVID-19 pandemic, face-to-face teaching in the module was replaced entirely with online lectures and tutorials. The students were engaged in individual and group activities to reinforce holistic cognitive skills. Activities included conducting a set of matrix presentations that relate problem statements with organism functions and how to solve them using biomimicry, weekly discussion of progress, and developing data reports. The introductory lectures by eminent ecologists and biomimicry experts with support from the university’s faculty members helped develop student knowledge and basic understanding of the systems in nature. 

#### 2.2.1. Student Project Design

The student project had three phases with two interim review sessions. Using the 3-M approach [3], educators created processes and tasks to help meet the three phases of project design (Table 1). The phases were graded, which constituted the overall grade of the module.

- Phase 1—Biomimicry Research

Students selected relevant biomimicry theories that allowed them to relate the attributes of the biological system to design problems and past examples from the literature. Students picked a research topic and generated a series of diagrams to illustrate their understanding of the theories on how natural elements work. Students submitted a report based on the findings.

- Phase 2—Problem Solving Proposal

Students proposed ideas and solutions reflecting the Problem Statement discussed in Phase 1. They identified the type of biomimicry—form, process, or system—and functions being mimicked through infographics and visuals to generate possible matrices. They also interviewed an expert in the chosen field and reflected on the critical and practical solutions of biomimicry.

- Phase 3—Prototyping through Biomimicry

Students continued to evaluate the proposed solutions by producing a prototype model/product/design that mimics nature with proposed matrices and configurations. Students documented the process of making the prototype and assessed performance results. The results were presented through posters/videos/images/calculations. 

#### 2.2.2. Student Learning Experience and Teaching Outcomes

The three-phase project design was an opportunity for the students to experience design-based learning. Design-based learning (DBL), or learning by making, is a learning method where students apply theory by creating and participating in design activities [20,21,22]. It is a learning strategy that is frequently linked with design and technology education to solve real-world problems through the construction of innovative and creative products [23].

It is well known that design-based learning can empower its learners through regular (e.g., weekly) engagements that lead to an effective understanding of cognitive and psychomotor skills. During phase 1, the students were able to experience how to define the challenge and biologize it. During phase 2, the focus was on discovery and abstraction that helped students reflect and think on the biomimicry design process. Phase 3 was learning through doing by creating prototypes, evaluating, and testing. 

Due to the blending of problem-solving activities and active learning during in-person and online classrooms, the activities resulted in an empowering engagement during class and created a meaningful learning experience.

## 3. Results and Findings

### 3.1. Thematic Analysis

**Figure 1:**
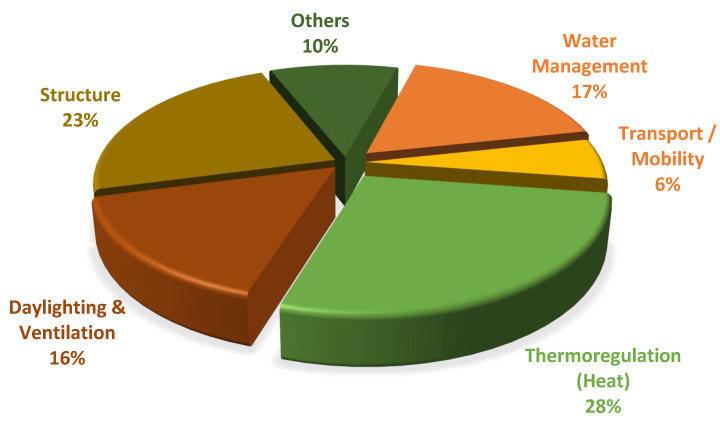
The frequency of student biomimicry projects classified by themes (*n* = 69).

The thematic analysis of student works from 2017 to 2022 led to five primary themes. Thermoregulation (28%) and structure (23%) were the most popular themes among students, followed by water management, daylighting and ventilation, and finally, transport/mobility (Figure 1). The “others” theme had varied topics related to acoustics, noise pollution, etc. Here, we showcase one student project per theme to give the readers a glimpse of the types of projects and their applications.

#### 3.1.1. Thermoregulation

Heat regulation is among the most significant of considerations when it comes to building design as the comfort of the inhabitants is a prime factor considered by architects while designing a building. The textures on an elephant’s skin can be mimicked to achieve thermal regulation and create sustainable building designs. 

**Figure 2:**
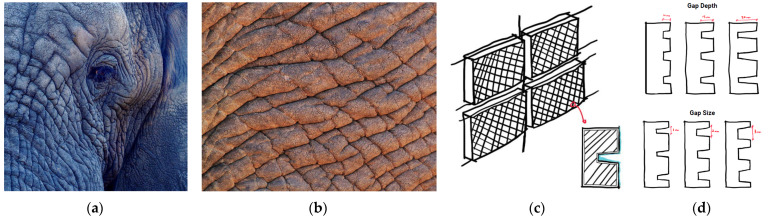
(**a**) Cracks on elephant skin. (**b**) Magnified view of cracks on the elephant skin. (**c**) Sketch mimicking the skin. (**d**) Sketch showing different widths and depths of the gaps for iterations. Photos: (**a**) Jennifer Latuperisa-Andersen Unsplash; (**c**,**d**) Photos: [24].

African and Asian elephants typically live in warm climates with average annual temperatures reaching up to 35 °C. Unlike humans, they lack sweat glands, which limits heat dissipation by sweating. However, elephants can thermoregulate through their textured skin. The bumps and crevices on the skin create shaded areas that limit heat gain. It also allows water retention through the skin that contributes to evaporative cooling and eventually heat dissipation (Figure 2). 

**Figure 3:**
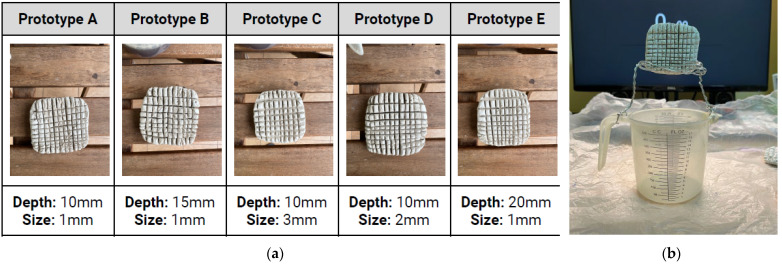
(**a**) Prototypes A to E were made from clay showing different depth and width of the gaps representing elephant skin texture. (**b**) Experimental procedure to test water retention capacity. Photos: [24].

These adaptations were mimicked by replicating the elephant skin pattern under different prototype models made of clay and testing them for their water retention capacity (see Figure 3, Figure S1). Experimental results showed the best gap size for water retention was 1 mm, and the best gap depth for reducing water flow speed was 15 mm. A direct application would be to recreate a building envelope surface with 1 mm gap size and 15 mm gap depth.

#### 3.1.2. Structures

**Figure 4:**
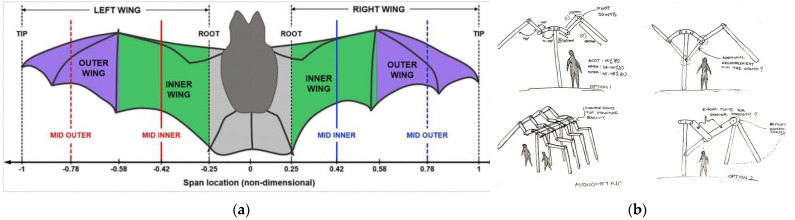
(**a**) The structural organization of a bat wing [25]. (**b**) A conceptual sketch mimicking bat wings. Photos: [26].

The bat wing is a modified mammalian forelimb, in which, over evolutionary time, the bones have become considerably lighter and certain bones have undergone elongation. Unlike the relatively rigid wings of insects and birds, a bat’s wing contains multiple joints overlaid by a thin elastic membrane (Figure 4, Figure S5). This gives bats extraordinary control over the three-dimensional shape their wings take during flight.

The flexibility of the bat wing was mimicked to create the blueprint of a temporary/mobile shelter or tent that can be erected in a short period of time between road alleys and be easily transported to different locations due to its lightweight design. It can also facilitate the treatment of medical emergencies or natural calamity relief measures.

**Figure 5:**
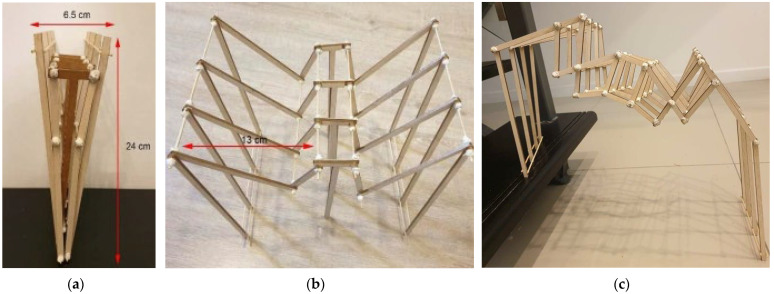
A prototype model based on a bat’s wing bone structure. (**a**) Double-span retracted position. (**b**) Double span (13 cm each side) with central support. (**c**) Single span up to 24 cm without central support. Photos: [26].

A double-span prototype model was created that can expand its structure up to 13 cm single span and retract to 6.5 cm (Figure 5; Figure S5). The structure was stable due to its double arm support design and multiple joints that add additional reinforcement to its central core strength. 

#### 3.1.3. Water Management

**Figure 6:**
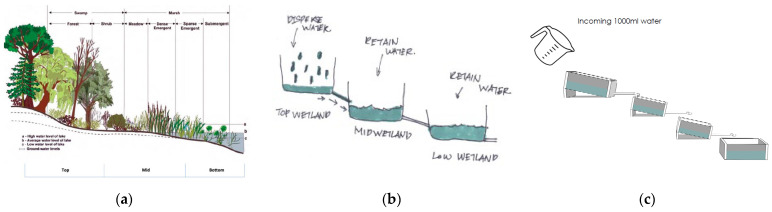
(**a**) Parts of a typical wetland. (**b**) Sketch replicating 3 wetland parts. (**c**) Sketch showing the experiment process. Photos: (**a**) [27], Illustrations: (**b**,**c**) [28].

A wetland is a distinct ecosystem that is flooded by water periodically or permanently. The presence of water largely influences the soil development and types of animal and aquatics plants living in the area. Wetlands typically have three parts with top wetlands comprising swamp forest and shrubs, mid wetlands comprising marshes, and bottom wetlands that interface the marsh to open waters (Figure 6a, Figure S3). The top and bottom parts are large in order to contain high intensity of rainfall, whereas the mid wetland is smaller in size to slow water flow and to create less ponding. 

**Figure 7:**
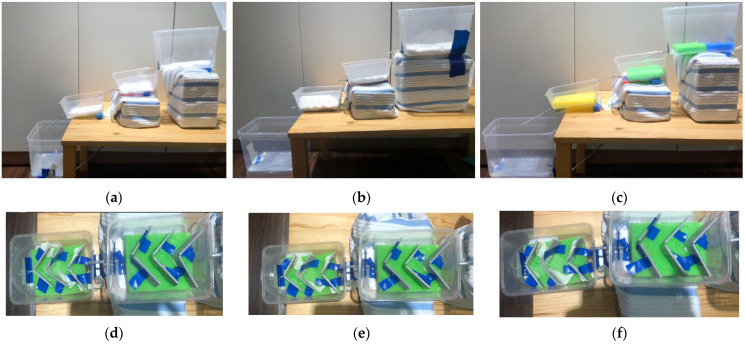
Prototype model testing different angles of wetlands and their water retention capacity. (**a**) 2° angle with a cotton pad; (**b**) 8° angle with cotton cloth; (**c**) 12° angle with a sponge; (**d**) 30° angle panels with 10 plicate leaves; (**e**) 50° angle panels with 13 plicate leaves; and (**f**) 70° angle panels with 15 plicate leaves. Photos: [28].

**Figure 8:**
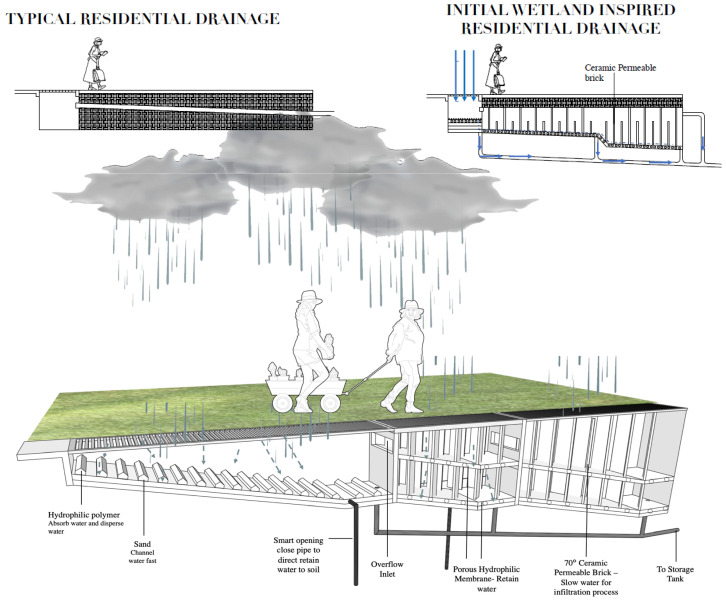
Comparison of typical residential drainage and the wetland inspired drainage to retain water and avoid flooding in residential buildings. Illustration: [28].

The wetland design was mimicked to create a biomimetic residential surface drainage system that can temporarily retain and control storm water runoff in the city of Kuala Lumpur (Figure 6b,c; Figure S3). This can serve to mitigate flash floods in tropical climates. The experiments aimed to identify the optimal slope, topology, and materials to be used for the proposed drainage system. Plastic containers were placed at different angles (2, 8, 12 degrees) with different materials (cotton pad, cloth, and sponge). The experimental results show that a 12° angle with a sponge aids faster run off from the first compartment and will be useful to avoid flooding. The cotton pads with smallest pore size aid in temporarily retaining water in the second and third compartment for optimum percolation into the soil (Figure 7). Moreover, the 70° angle panels slow water for increased infiltration rate and 15 plicate leaves help to disperse incoming water. Mimicking such a wetland system can better control water flow, retain, and slowly release water to the storage tank compared with the typical residential storm water drainage system (Figure 8).

#### 3.1.4. Daylighting and Ventilation 

**Figure 9:**
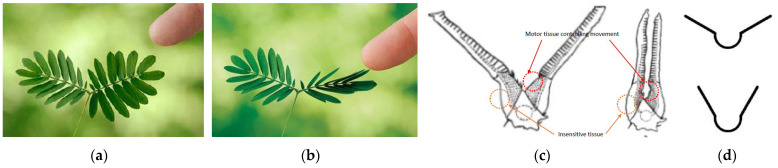
*Mimosa pudica* plant leaflets: (**a**) normal position, (**b**) closing position when touched. (**c**) Rapid movement controlled by motor tissue. (**d**) Line sketch mimicking the plant. Photos: (**a**,**b**) [29], (**c**) [30], and (**d**) [31].

*Mimosa pudica*, also known as the touch-me-not plant, is a creeping annual or perennial herb that belongs to the pea family—Fabaceae (Leguminosae). This sensitive plant is well known for its rapid leaf movements in response to stimuli such as touch, temperature, sunlight, and vibration. Figure 9 shows the rapid folding movement that happens when the leaflets are stimulated due to changes in turgor pressure caused by vibrations (also see Figure S4). The closing and opening of leaflets are controlled by the pulvinus motor tissue (present at the base of the leaf), which loses turgor pressure when stimulated.

**Figure 10:**
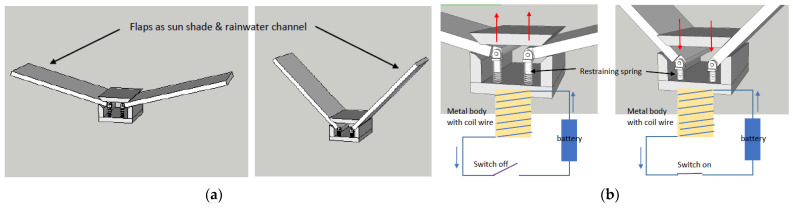
(**a**) Opening and closing of the shading device. (**b**) Electromagnetic circuit controlling the movement of the shading device [31].

A sun shading device was designed to shade the building facades by mimicking the rapid movement of leaflets. To assimilate the opening and closing of leaflets by its motor tissue, the operation of the sun shading device was controlled by an electromagnetic system with restraining spring as shown in Figure 10 (also see Figure S4). Under normal conditions, the electromagnet circuit turns off and the closed flaps act as a sun shading device. Under shady conditions, the circuit can be turned on, causing the spring to contract, and the flaps will be opened to allow natural light to enter the space. Once the circuit is turned off, the restraining spring is released, returning the flaps to their original position.

#### 3.1.5. Transport/Mobility

**Figure 11:**
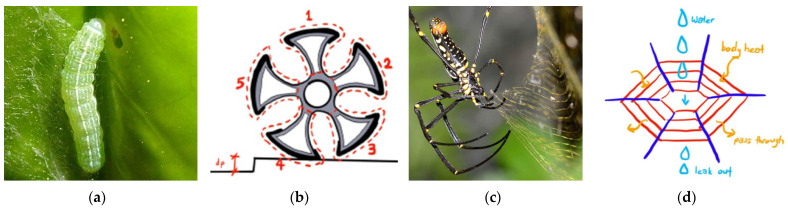
(**a**) Leaf roller caterpillar. (**b**) Sketch of a wheel mimicking leaf roller. (**c**) Golden orb web spider *Nephila pilipes.* (**d**) Sketch of a woven pattern mimicking the spider web patterns. Photos: (**a**) [32], (**c**) [33], Illustrations: (**b**,**d**) [34].

Leaf rollers are small greenish to green/brown caterpillars (moth larvae) that feed on leaves, buds, and fruit. Leaf roller caterpillars move around by inching or crawling (Figure 11a; Figure S2). Their ability to navigate on branches makes them particularly interesting bio-mechanical subjects. The golden orb web spider (*Nephila pilipes*) creates a web with a major ampullate silk that has high tensile stress and little elasticity at the center (Figure 11c; Figure S2).

**Figure 12:**
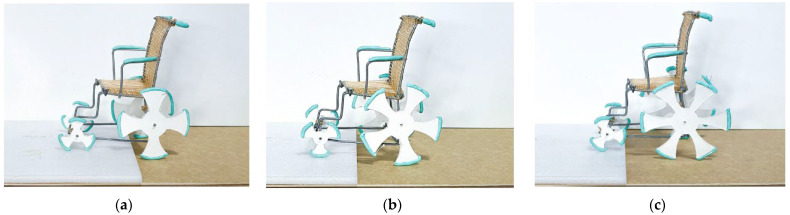
Prototype models with experimental iterations. (**a**) Four-leaf, 20 inches wheel. (**b**) Five-leaf, 22 inches wheel. (**c**) Six-leaf, 24 inches wheel. Photos: [34].

The functions of the leaf roller caterpillar and golden orb web spider were mimicked in the wheelchair design. The design of wheels mimicked the design of leaf roller caterpillars’ prolegs that help its movement by inching or crawling. The web structure of golden orb web spider was adapted for the seat and back support of the wheelchair. The web structure keeps the seat strong, lightweight, and breathable. Figure 12 shows the iterations matrix and the best-performing model to aid disabled people to move from one place to another with minimal help from others (also see Figure S2). 

The results showed that the six-leaf, 24 inches diameter wheel performed better in terms of smooth movement and usage in wet areas compared to other wheel dimensions and number of leaves.

#### 3.1.6. Synthesis 

**Figure 13:**
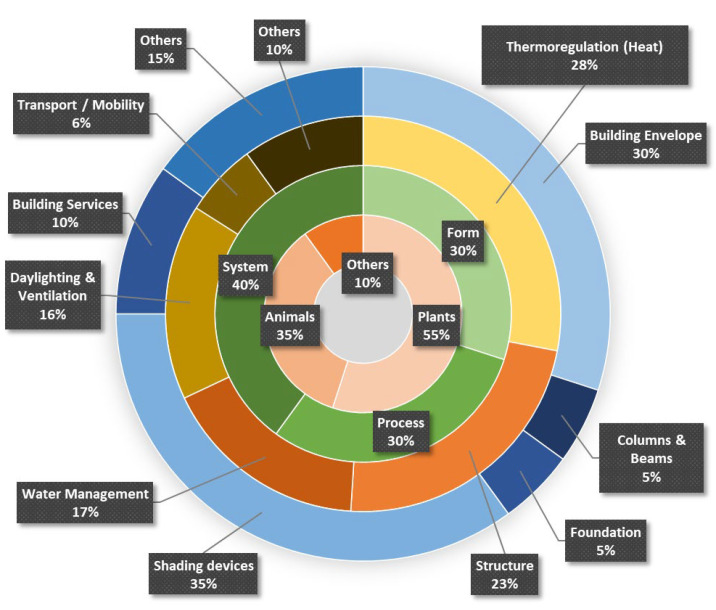
Classification of student projects by the type of model organism, type of biomimicry, themes, and biomimicry application at different levels.

The synthesis of thematic analysis is presented in a wheel diagram with bands representing the classification of student projects at various levels (Figure 13). The percentages in Figure 13 elucidate the frequency of projects under each category. In the first wheel, the students identified the organisms such as plants, animals, or other life forms that were identified as model organisms. The second wheel identified the type of biomimicry, i.e., form, process, or system. The third wheel identified the function in terms of heat, light, water, structure, and others that were mimicked. Finally, the area of application was identified in the fourth wheel. This included areas such as building envelopes, columns, beams, foundations, shading devices, building services, and others. 

The results show that students preferred choosing plants (55%) as model organisms over animals and other life forms. The type of biomimicry was divided between form (30%), process (30%), and system (40%) examples. Mimicking the thermal strategies of organisms was more popular (28%) than mimicking other functions. When it comes to application, shading devices were the most attractive (35%) followed by building envelope, services, etc.

### 3.2. Student Feedback

Twenty-eight responses were received for the online survey questionnaire that was distributed to the Nature and Architecture elective module alumni. Their feedback is presented in the following paragraphs.

#### 3.2.1. Reasons to Choose the Module

**Figure 14:**
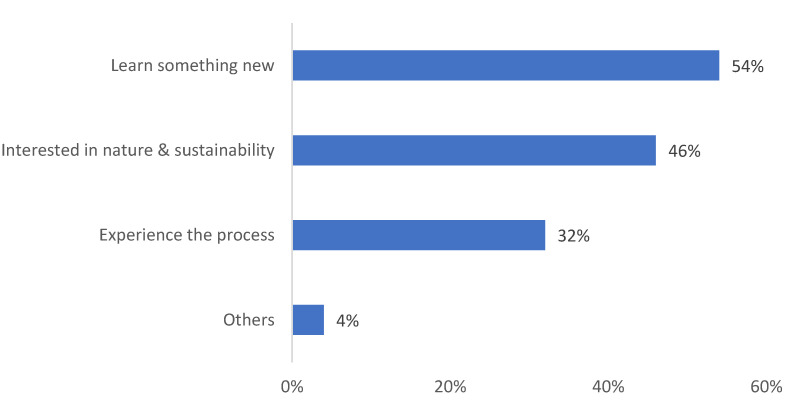
Student responses to the reasons for choosing the N&A module.

The majority of the respondents (54%) mentioned that they chose the module because they wanted to learn something new and because they have an interest in nature and sustainability (46%) (see Figure 14).

#### 3.2.2. Impact of the Module on Student Approach and Thinking

**Figure 15:**
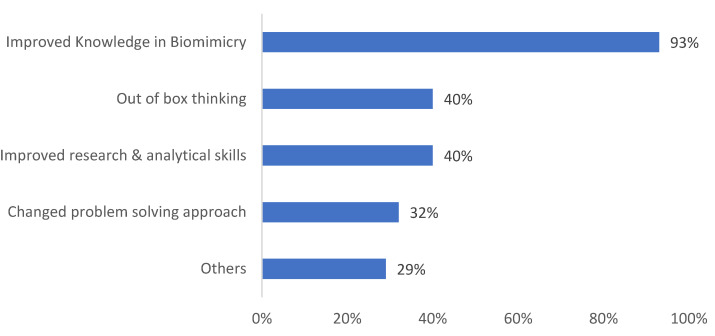
Student responses to how the N&A module changed their approach and thinking.

In order to understand the impact of the module on the student’s thinking, the question *“In what way did this module change your approach and thinking?”* was posed. Most of the students (93%) acknowledged that the module had changed their approach and thinking. It not only improved their knowledge of biomimicry and sustainability but also their research and analytical skills (see Figure 15). 

#### 3.2.3. Ways to Improve the Module

**Figure 16:**
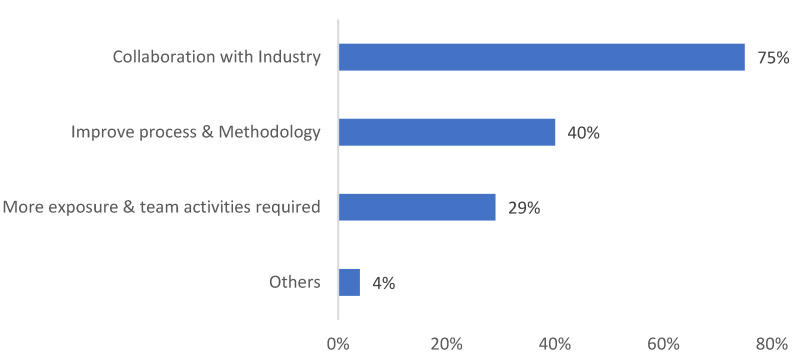
Student responses to the question on ways to improve the N&A module.

However, when asked the question “*How can we improve the module?*”, most of the students (75%) suggested that the module could be improved by having better collaboration with the industry (Figure 16).

Some students who studied during the COVID-19 pandemic when the module was conducted entirely online highlighted the importance of face-to-face learning, particularly in a natural setting:

“*Experience nature as it is: We can’t learn nature much through online classrooms and imagine (brain simulation)—we should visit nature places, to collect realistic data in nature, design and test things in nature.*”

The students also highlighted the importance of interdisciplinary learning. Since biomimicry design needs a deeper understanding of biology, a collaboration with ecology or biology students would be beneficial:

“*Everything is connected in nature: more collaboration with ecology/biology students or specialists, or more group work among students.*”

#### 3.2.4. Industry Application

**Figure 17:**
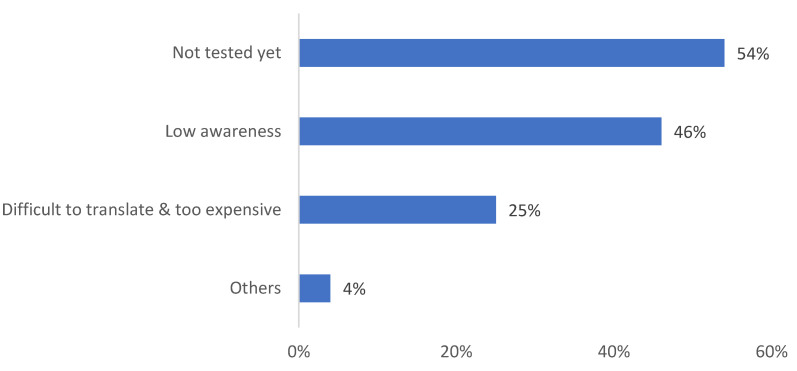
Student response to challenges of applying biomimicry design to the industry.

To understand on how much the module alumni applied their biomimicry design knowledge and skill at their work, a question *“What were some challenges in applying the learnings of this module to industry?”* was posed. The majority of the students (54%) said that they had not tested the learnings from the module yet (see Figure 17). Many also mentioned that there is still low awareness (46%) about biomimicry design in the architecture industry in Malaysia. Furthermore, 25% of the alumni found it difficult to translate research to real design and felt the process was too expensive.

### 3.3. Innovation Competition (Innofest)

The student works from the module were submitted for the Innovation Competition of Taylor’s University (Innofest) of March 2021, August 2021, and March 2022. Taylor’s Innofest is an innovation festival digital platform showcasing ongoing design thinking, creativity, and direct research and development projects undertaken by undergraduate and postgraduate students in the university. This event aspires to promote the spirit and culture of creativity and innovation throughout the university, showcasing students works that provoke discussion, stimulate ideas, and collaborations and aligning research outputs to Technology Readiness Level 1 to 9. Projects with market potential and/or social value that solve macro- or micro-economic or social challenges are identified in the competition. These project teams are mentored and given an opportunity to scale up, pitch to investors, transfer technology, productize, collaborate, and/or go to market via spin-offs or start-ups.

The student works from the Nature and Architecture module received the following prizes:

- August 2021—Third Prize, Best Potential Intellectual Property Right Filing (Pinecone-scale-inspired rainwater harvester).
- March 2022—First Prize, Best Potential Intellectual Property Right Filing (*Mimosa pudica* innovative folding system), and First Prize, Taylor’s University Commercialization Potential (*Mimosa pudica* innovative folding system).

The winners were given a chance to enter the Taylor’s University internal innovation funding program known as IGNITE 10′ s Minimum Viable Product category and SOCIAL 10 Social Innovation category. Seed funding was also made available to exceptional projects, alongside mentoring provided by Taylors Me.reka Makerspace on prototyping, design thinking, feature development, and BIZPOD on business planning, incubation, fund raising, and industry partnerships.

## 4. Discussion

### 4.1. Diversity of Student Works and Preferred Themes

Our findings highlight the diversity of student-led innovations possible in a tropical climate. Some model organisms and/or systems that students emulated included the elephant, bat, wetland, leaf roller caterpillar, and golden orb web spider that are native to Malaysia and the touch-me-not (*Mimosa pudica*) shrub that is not native but naturalized and commonly found in southeast Asia. While plants comprise only about a quarter of known species [35], it was surprising to find out that students preferred plants as model organisms over animals and other life forms. Students are likely to choose model organisms that are suited for their challenge, found in their climate, and/or for which sufficient life-history research is available that would make it possible to emulate their strategies. The megadiverse tropical Malaysia and southeast Asia at large provide a rich catalog of model organisms for architectural students and practitioners to choose from. This should be of interest as our study describes student works from one of the few biomimicry in architecture modules taught in a university in southeast Asia and possibly the wider tropical region. Thermoregulation and structure making were the most popular biomimicry challenges selected by the students, which was followed by water management, daylighting and ventilation, and transport and mobility. These challenges are likely to be reflective of architectural student and practitioner priorities for design in the tropics. Better thermoregulation, daylighting, and ventilation in buildings for example can lead to superior energy performance and reduction in carbon emissions. 

### 4.2. Reflections on Student Learning, Quality, and Industry Readiness

An analysis of alumni questionnaire survey feedback points to mixed outcomes. The N&A module improved the student approach and thinking based on new knowledge gained in biomimicry. It also demonstrated significant improvement in students’ knowledge related to natural theories, cognitive skills, and self-efficacy related to issues relating to sustainability in the built environment.

The immersive learning experience adopted in the module seemed to benefit the students, except during the COVID-19 pandemic when students craved more in-person engagements. Moreover, some students were quick to highlight that biomimicry theory and discovery phases would be done better outdoors in nature than in indoor classrooms or over online sessions. 

The prototype models developed by students were generally reflective of their ability and understanding of biomimicry applications. They were typically built using cost-effective materials and more often than out, conceptual than being ready for real-life testing. One way to improve the quality and accuracy of the prototypes would be to push the students to conduct more intensive design simulations (prior to developing prototypes) as shown by similar modules elsewhere (see [19,36]). This would ensure that that the final prototype developed by the students is closer to industrial application and has a higher chance of commercialization.

We also learned that the students faced numerous challenges in adhering to the biomimicry design process due to time limitations in the module. The students remarked during their feedback that they only developed sufficient understanding of the biomimicry design process during the prototype development stage. This suggests that it would be beneficial to introduce the concepts and applications of biomimicry in a separate module earlier during the master’s or undergraduate degree. 

### 4.3. Industry Engagement

It is also clear that the adoption of biomimicry in industry applications has many challenges that need to be overcome through increased awareness of this discipline and potential incentive/subsidy schemes that could help the adoption of biomimicry ideas to industry. We recommend better integration of the module with the industry by greater industry collaboration and, thereby, exposure of students to practical applications and limitations. 

Meanwhile, we believe that the talented students trained in biomimicry design by Taylor’s University can play an important part in advancing biomimicry efforts in the industry and drive Malaysia’s sustainability ambitions. Malaysia currently ranks 65 out of 165 countries in its sustainable development goal index [37]. There is high national interest and commitment toward sustainability by increasing literacy in this field. The student feedback reinforced the fact that students benefitted from the module, and this will lead to building capacity for sustainability professionals in the built environment in Malaysia.

## 5. Conclusions

The motivation behind this publication was to demonstrate the diversity of innovations developed by the N&A module students across challenges and themes (e.g., thermoregulation, structures) and biomimicry model organisms particularly as the module is based in a megadiverse tropical climate where the students are spoilt for choice for model organisms suited to the tropical climate and context. The underlying motivation was also to take stock of the key elements of the module (its philosophy, preferred student themes, and learning outcomes), evaluate the module’s strengths and limitations, and improve its delivery for a more in-depth understanding and engaging delivery for the students. This was achieved through a robust understanding of student feedback and issues. We managed to showcase the experiential learning of the students and the challenges they faced in applying biomimicry to the industry upon graduation. Several future directions are highlighted such as an early introduction of biomimicry theory to interested students and upskilling the industry.

## Figures and Tables

**Table 1:** Relationships between biomimicry design process (based on [3]) and teaching/learning activities and learning outcomes.

Phases and 3M Approach	Biomimicry Design Process	Teaching and Learning Activities	Expected Outcome
Phase 1: Nature as Model	Define the challenge	Introduction (problem statement, aim, and RQ) reflecting context. Background study (selected biomimicry theories, structure, and system). Propose solutions through proposed ideas and options reflecting the chosen problem statement.	Introduction (problem statement, research question, and aim) reflecting the climate challenges. Background study (selected biomimicry theories, structure, and system).
	Biologize function and context	Utilize flexible and divergent thinking through the demonstration of form, function, process, and system from detailed research reflecting problem statement.	Students produce a series of slides illustrating the research findings, analysis on the theories, and its outcome.
Phase 2: Nature as Mentor	Discover biological strategies	Communicate information and research through proposed ideas and solutions reflecting the problem statement.	Proposed ideas and solutions reflecting the problem statement. Discuss the form, function, process, and system through infographics/visuals.
	Abstract design strategies	Recognize and value the discussion through the demonstration of form, function, process, and system through infographics/visuals.	Produce a report providing the studies and proposals of the above components. A series of analysis diagrams and options must be arranged and presented through a visual report.
Phase 3: Nature as Measure	Emulate nature’s lessons	Generate ideas from nature for their designs solutions. Demonstrate clear research method and methodology. Produce prototype model/products/design that mimics nature.	Generate ideas from nature for their designs solutions. Demonstrate clear research method and methodology. Produce a prototype model/products/design that mimics nature.
	Evaluate fit and function	Clarity of specification of all components studied with good references and narration.	Students will exhibit their findings, analyze the required submission as explored in Part 1 and 2, and show its integration of the identified system in their final prototype.

## Data Availability

Not applicable.

## Supplementary Materials

The following supporting information can be downloaded at https://www.mdpi.com/article/10.3390/biomimetics8010013/s1: Figure S1 Student poster on mimicking the elephant skin texture facades for thermoregulation [24], Figure S2 Student poster on mimicking the Leaf Roller and Orb Web Spider *Nephilla pilipes* for better transport and mobility [34], Figure S3 Student poster on mimicking wetlands for better water management [28], Figure S4 Student poster on mimicking *Mimosa Pudica* leaves for better daylighting [31], Figure S5 Student poster on mimicking Bat’s Wing for temporary structure [26].
