# Supplementary material for: Teaching Nature and Architecture: Student-Led Account of Biomimicry Innovations in the Tropics
Source: Biomimetics (Basel). 2023 Jan 1;8(1):13. doi: 10.3390/biomimetics8010013 (PMC9844408; doi:10.3390/biomimetics8010013)
Supplement: Supplementary file 1 [file biomimetics-08-00013-s001.zip › biomimetics-2099146-supplementary.pdf]

biomimetics

MDPI

Supplementary Materials

Nature & Architecture: Student-led Biomimicry Innovations in the tropics

Girirajan Arumugam [1], Siti Norzaini Zainal Abidin [1], Camelia May Li Kusumo [1] and Anuj Jain [1,2,*]

[1] School of Architecture, Building and Design, Taylor's University, Malaysia; giribarch@gmail.com (G.A.); sitinorzaini.zainalabidin@taylors.edu.my (S.Z.); cameliamayli.kusumo@taylors.edu.my (C.K.)
[2] Biomimicry Singapore Network and bioSEA Pte. Ltd., 68 Chestnut Avenue, Singapore; anuj@biosea.sg (A.J.)
* Correspondence: anuj@biosea.sg (A.J.)

SUPPLEMENTARY MATERIALS

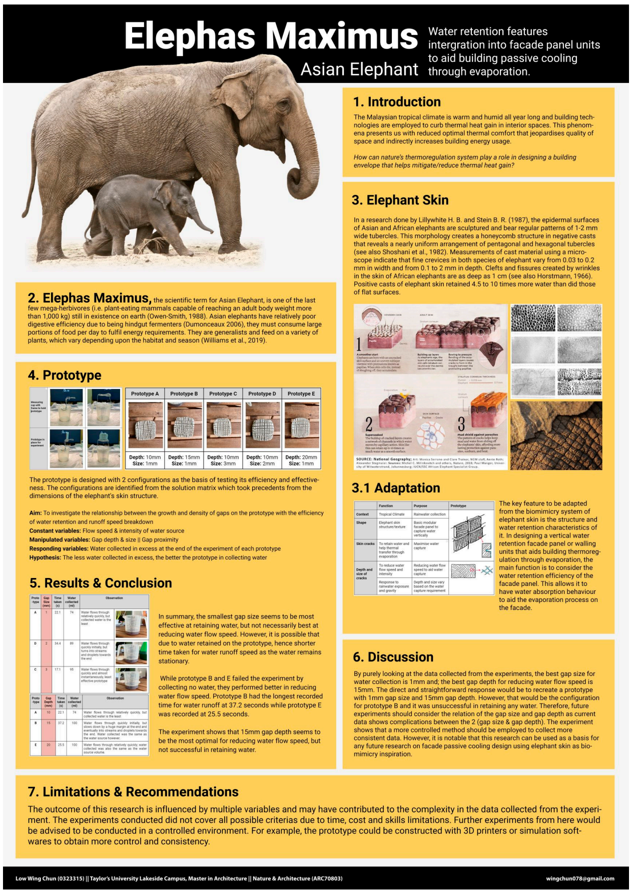

Figure S1: Student poster on mimicking the elephant skin texture facades for thermoregulation - (Chun, 2022)

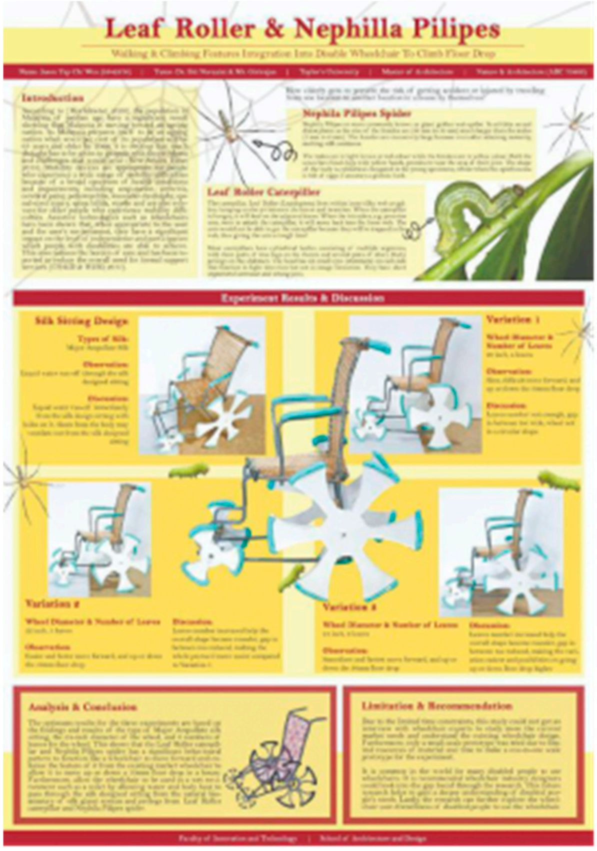

Figure S2: Student poster on mimicking the Leaf Roller and Orb Web Spider *Nephilla pilipes* for better transport and mobility - (Wen, 2022)

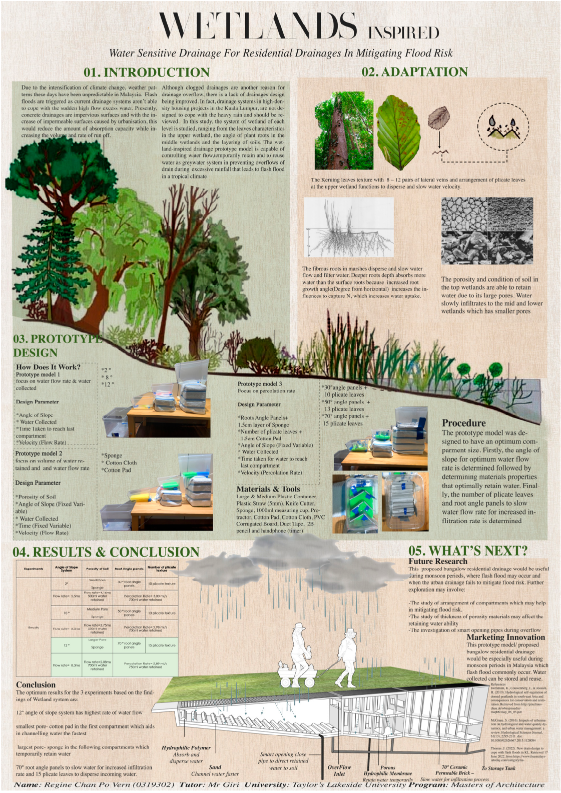

Figure S3: Student poster on mimicking wetlands for better water management - (Vern, 2022)

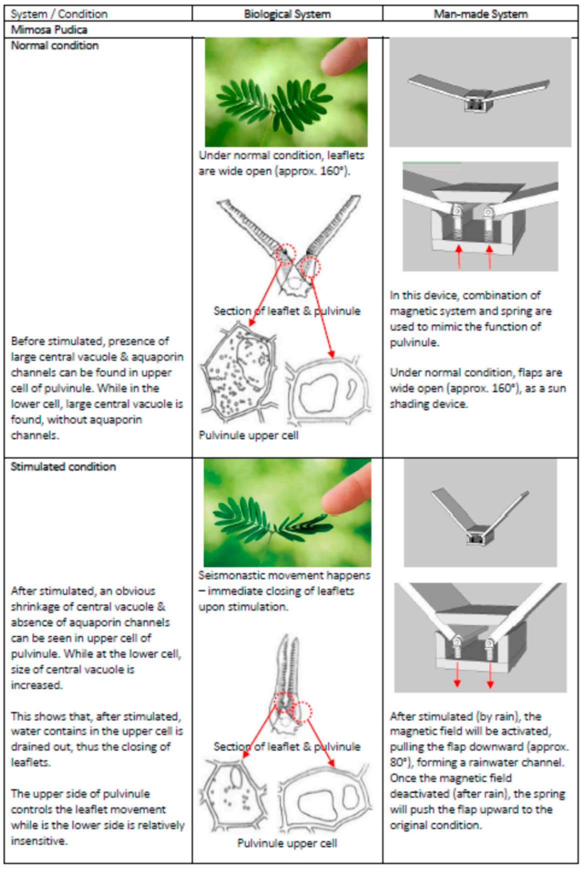

Figure S4: Student poster on mimicking *Mimosa Pudica* leaves for better daylighting - (Yin, 2017)

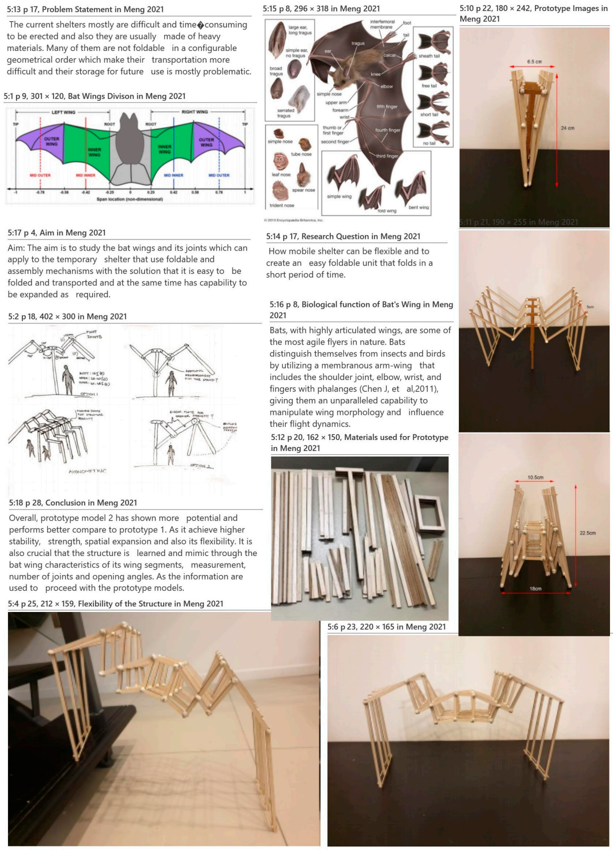

Figure S5: Student poster on mimicking Bat’s Wing for temporary structure - (Meng, 2021)
